# Concurrent COVID-19 and Acute HIV: A Case Report and Diagnostic Review

**DOI:** 10.1155/2021/2653678

**Published:** 2021-11-16

**Authors:** Kelly A. Johnson, Sally Graglia, Elizabeth D. Lynch, Joanna De Mesa, Erin Antunez, Sandra Torres, Susa Coffey, Stephanie E. Cohen

**Affiliations:** ^1^University of California San Francisco, Department of Medicine, Division of Infectious Diseases, San Francisco, CA, USA; ^2^San Francisco Department of Public Health, Population Health Division, San Francisco, CA, USA; ^3^University of California San Francisco, Department of Medicine, Division of HIV Infectious Diseases and Global Medicine at Zuckerberg San Francisco General Hospital, San Francisco, CA, USA; ^4^University of California San Francisco, Department of Emergency Medicine, San Francisco, CA, USA; ^5^HealthRIGHT 360 Clinic, San Francisco, CA, USA

## Abstract

A 26-year-old male presented to the emergency department feeling unwell in February of 2021 with symptoms including diaphoresis, loose stools, and loss of taste sensation. Workup not only confirmed a diagnosis of COVID-19 but also revealed discordant HIV test results, with a reactive fourth-generation antigen/antibody test but a negative HIV-1/2 differentiation immunoassay. Subsequent HIV viral load testing obtained two days later ultimately established a diagnosis of acute HIV (AHI). Screening for HIV and other sexually transmitted infections decreased during the COVID-19 pandemic. It is critical that providers (1) continue recommended screening for HIV as an essential service; (2) consider acute HIV in the differential when evaluating patients with acute viral syndromes; (3) recognize that AHI can occur concurrently with other infections, including COVID-19; and (4) understand the differential diagnosis for discordant HIV test results and know when HIV viral load testing is needed to resolve such discordant results.

## 1. Introduction

With over 33 million cases and nearly 600,000 deaths in the United States alone, [1] COVID-19 remained at the forefront of attention for both public health and clinical medicine throughout much of 2020 and into 2021. Because COVID-19 was an extremely common diagnosis during this period, this illness was often at the top of the differential when patients presented with nonspecific symptoms suggestive of viral illness. Non-COVID-19 diagnoses such as acute HIV (AHI) with similar presentations could have been easily missed. In this case report, we discuss a case of acute HIV (AHI) diagnosed concurrently with COVID-19. To our knowledge, this is the first detailed report of COVID-19 and AHI coinfection. Our goals are to (1) describe and draw attention to the symptom overlap between viral illnesses such as COVID-19 and AHI, (2) review the interpretation of discordant HIV test results with a focus on AHI, and (3) encourage providers to continue screening for HIV in routine clinical settings even during public health crises such as the COVID-19 pandemic.

## 2. Case Presentation

A 26-year-old male presented to the emergency department (ED) in February of 2021 feeling generally unwell, with 3 days of symptoms including diaphoresis, dizziness, loose stools, and loss of taste sensation. His past medical history included occasional methamphetamine and cocaine use along with attention-deficit/hyperactivity disorder. Vital signs included a temperature of 36°C, heart rate of 90 beats per minute, blood pressure of 131/80 mm of mercury, and oxygen saturation of 97% on room air.

Bloodwork revealed a white blood cell count of 6.6 × 10^9^ cells/liter with lymphopenia (absolute lymphocytes 0.3 × 10^9^ cells/liter). The patient's complete blood count and comprehensive metabolic panel were otherwise normal. Initial evaluation for pulmonary and gastrointestinal infections included a chest X-ray which demonstrated clear lungs and negative stool polymerase chain reaction (PCR) testing for *Clostridium difficile*. A nasopharyngeal PCR swab confirmed a diagnosis of COVID-19.

The ED physician took a sexual history, noting that the patient had recently ended a relationship with a female partner and had several condomless sex partners thereafter. The patient was screened for HIV in the ED, with results as presented in Table 1. He was discharged with these results still pending. A laboratory-based 4^th^-generation HIV antigen/antibody (Ag/Ab) test was reactive, while HIV differentiation testing was negative. Outpatient HIV testing was repeated two days later, this time including an HIV RNA. The patient was started on antiretroviral therapy (ART) while awaiting his repeat test results. Again, the 4^th^-generation Ag/Ab test was reactive, and the differentiation test was negative. HIV RNA was detectable at nearly 1 million copies/ml. A diagnosis of AHI was confirmed and disclosed to the patient, who continued ART and immediately established HIV care.

## 3. Discussion

This case of a young man with concurrent COVID-19 and AHI (defined as the initial weeks after HIV infection, prior to antibody formation) illustrates several important points regarding the presentation and diagnosis of AHI, especially during a global public health crisis such as the COVID-19 pandemic.

First, likely due in part to COVID-19-related disruptions in routine clinical services, screening for HIV declined dramatically during the COVID-19 era. Compared with the same period in 2019, following implementation of stay-at-home or shelter-in-place orders throughout much of the country, there were nearly 700,000 fewer screening HIV Ag/Ab tests sent and almost 5,000 fewer new HIV-1 diagnoses confirmed at a large U.S. commercial laboratory from March–September 2020 [2]. While the Centers for Disease Control and Prevention (CDC) recommends that all people aged 13–64 be screened for HIV at least once (and that people with risk factors be screened at least annually), these recommendations were likely deferred throughout much of 2020-2021. At the same time, HIV preexposure prophylaxis (PrEP) use and uptake declined, with modeling studies estimating a 21% decrease in PrEP prescriptions and a 28% decrease in new PrEP users in March–September of 2020 compared with the prior year [3].

Screening for other sexually transmitted infections (STIs) similarly decreased during the COVID-19 public health emergency. Nationally reported cases of gonorrhea (GC), chlamydia (CT), and primary/secondary syphilis declined steeply in March 2020, despite having risen steadily over the last ten years, resulting in over 27,000 fewer reported GC cases than would have otherwise been expected in April of the same year [4]. U.S. cities and states demonstrated similar findings: the state of California, for example, reported 31%, 15%, and 13% fewer cases of CT, primary and secondary syphilis, and GC in January–June 2020 compared with the same period in 2019 [5].

These declines in HIV/STI diagnoses were unlikely to have been driven exclusively by COVID-19-related changes in sexual behavior. While some patients may have changed their sexual practices in response to social distancing requirements, many likely did not, especially as COVID-19 continued into a second year. A review incorporating 20 articles from 12 countries confirmed that many people (25–60%) did not reduce their numbers of sexual partners during the COVID-19 era [6]. In at least one U.S. survey of men who have sex with men, respondents instead reported mean increases of 2.1 anal sex partners [7].

For these reasons, many patients remained vulnerable to STIs/HIV but were less likely to be screened in routine clinical settings at the height of the COVID-19 pandemic. Knowing that patients may have missed routine HIV screening, it is imperative for clinicians to consider HIV in their differential diagnoses for viral syndromes, particularly since, as in the case of our patient, there can be a significant symptom overlap between AHI and other viral processes. Indeed, many of the classic COVID-19 signs/symptoms [8], including fever, myalgias/arthralgias, pharyngitis, and even gastrointestinal symptoms, are nonspecific and can also be seen in AHI [9]. In terms of laboratory abnormalities, lymphopenia may similarly be observed in both conditions [10, 11]. Our patient's AHI diagnosis could have easily been missed had his symptoms been ascribed exclusively to COVID-19 and HIV screening not been sent. Without a confirmed AHI diagnosis, the patient would have been unable to rapidly start ART, an approach known to decrease the risk of forward transmission while reducing loss-to-follow up, improving virologic suppression rates, and potentially decreasing mortality [12].

Providers should resume screening for HIV and other STIs as an essential service and should consider AHI when evaluating patients like ours with acute viral syndromes. We were fortunate that a sexual history was taken and that HIV screening was initiated when our patient first presented to the ED. Such steps could easily have been deferred, particularly in a busy clinical setting and with an already-confirmed COVID-19 diagnosis. The benefits of sending workup for HIV alongside COVID-19 were similarly demonstrated in Chicago, where a 2020 initiative incorporating routine HIV screening for all patients undergoing COVID-19 evaluations resulted in 9 diagnoses of AHI, including one other case of AHI/COVID-19 coinfection, the details of which have not been previously described in the literature [13].

In addition to remaining vigilant to AHI as an alternative or concurrent explanation for nonspecific viral-like symptoms, providers should consider AHI as a possibility when interpreting discordant HIV test results rather than assuming such results represent false positives. After initial HIV exposure, HIV RNA becomes detectable first (often within days of infection), followed by the p24 antigen (detectable within 2-3 weeks after exposure) and finally HIV-1 and 2 antibodies (detectable another week or more thereafter). In routine clinical settings, the Centers for Disease Control and Prevention recommends [14] screening for HIV with a laboratory-based HIV Ag/Ab combination immunoassay, capable of detecting the p24 antigen as well as HIV-1 and 2 antibodies [15]. If the laboratory-based HIV Ag/Ab test is reactive, as was the case with our patient, the second recommended step in workup is a separate immunoassay differentiating antibodies to either HIV-1 or HIV-2. In cases where the differentiation assay is positive for such antibodies, a diagnosis of HIV is confirmed. However, if the differentiation is indeterminate or negative, as was seen with our AHI patient (who presumably had a detectable p24 antigen but had not yet formed HIV antibodies), an HIV RNA is required as the final diagnostic step [14].

**Figure 1:**
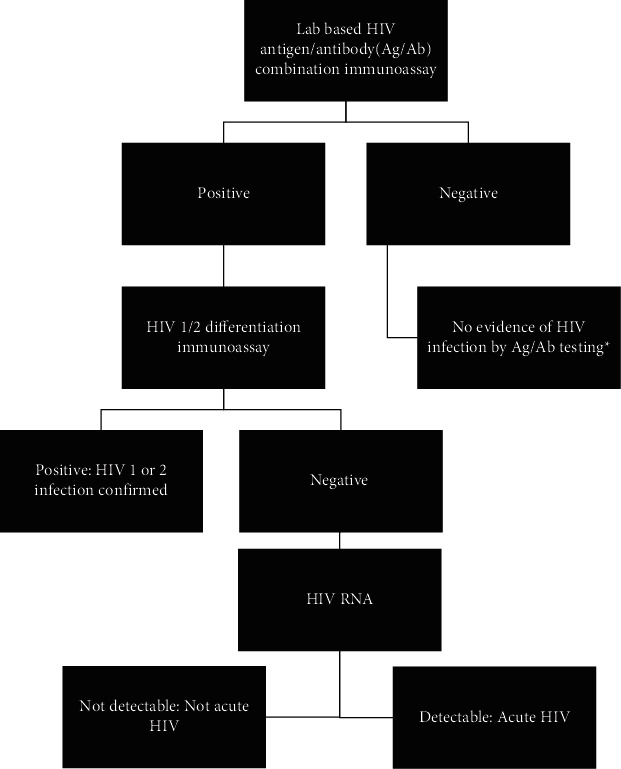
Recommended HIV screening algorithm in routine clinical settings. ^∗^HIV viral load testing still indicated if high clinical concern for acute HIV and/or in high incidence patient populations, as this is the first test to become positive after HIV acquisition.

In line with this recommended HIV testing algorithm (Figure 1), our patient had repeat HIV testing, including an HIV RNA, performed within two days of his initial discordant HIV test results. This process allowed for rapid confirmation of the AHI diagnosis and linkage to HIV care. Such favorable outcomes would not have been possible had this patient not been (A) initially screened for HIV/AHI alongside COVID-19 and (B) rapidly retested with an HIV RNA in response to his initial HIV test results. While viral load testing is rarely used as a screening tool due to cost and turnaround time, providers should consider sending an HIV RNA alongside initial Ag/Ab testing in cases where there is a high suspicion for AHI based on symptoms or exposures, as the former test can be necessary to clinch an AHI diagnosis.

To our knowledge, this is the first detailed description of a case of concurrent AHI and COVID-19. This case highlights the importance of recognizing the symptom overlap between AHI and other viral illnesses, reinstating routine HIV screening practices, and using HIV RNA testing when needed to resolve discordant HIV test results.

## Figures and Tables

**Table 1:** HIV test results in a case of concurrent acute HIV and COVID-19.

	Initial testing	Subsequent testing (obtained 2 days later)
4^th^-generation HIV antigen/antibody^*∗*^	Reactive	Reactive
HIV differentiation assay^*∗∗*^	Negative	Negative
**HIV viral load**	**Not sent**	**999,908 copies/mL**
Absolute CD4 count	N/A	172 cells/mm^3^ (33%)

^*∗*^Abbott ARCHITECT HIV Ag/Ab combo. ^*∗∗*^Bio-Rad Geenius HIV-1/2 confirmatory assay.

## Data Availability

No data were used as this case report involves a single patient.
